# Integration of care systems in Portugal: anatomy of recent reforms

**DOI:** 10.5334/ijic.989

**Published:** 2014-07-24

**Authors:** Silvina Santana, Nina Szczygiel, Patrícia Redondo

**Affiliations:** Institute of Electronics Engineering and Telematics of Aveiro (IEETA), University of Aveiro, Aveiro, Portugal; Department of Economics, Management and Industrial Engineering, University of Aveiro, Aveiro, Portugal; Department of Economics, Management and Industrial Engineering, University of Aveiro, Aveiro, Portugal; Department of Economics, Management and Industrial Engineering, University of Aveiro, Aveiro, Portugal; Hospital Infante D. Pedro, Aveiro, Portugal

**Keywords:** care systems integration, health care reform, social care reform, Portugal

## Abstract

**Background:**

Integrated care is increasingly present in the agenda of policy-makers, health professionals and researchers as a way to improve care services in relation to access, quality, user satisfaction and efficiency. These are overarching objectives of most sectoral reforms. However, health care and social care services and systems are more and more dependent on the performance of each other, imposing the logic of network. Demographic, epidemiologic and cultural changes result in pressure to increase efficiency and efficacy of services and organisations in both sectors and that is why integrated care has become so relevant in the last years.

**Methods:**

We first used concept maps to organise and systematise information that we had gathered through deep literature review in order to set a framework where to base the subsequent work. Then, we interviewed informants at several levels of the health and social care systems and we built a list of major recent reforms addressing integrated care in Portugal. In a third step, we conducted two independent focus groups where those reforms were discussed and evaluated within the context of the concepts and frameworks identified from the literature. Results were confronted and reconciled, giving place to a list of requisites and guidelines that oriented further search for documentation on those reforms.

**Results:**

Several important health reforms are in course in primary and hospital care in Portugal, while a so-called third level of care has been introduced with the launch of the National Network of Long-Term Integrated Care (RNCCI – *Rede Nacional de Cuidados Continuados Integrados*). The social care sector has itself been a subject of alternative models springing from opposite political orientations. All these changes are having repercussions on the way the systems work with each other as they are leading to ongoing and ill-evaluated reformulations on the way they are governed, financed, structured and operated.

**Conclusions:**

Care integration is not absent from policy-making and implementation endeavour in Portugal. However, recurrent issues seem to be consistently hampering the efforts regarding the integration of care in the country. It is urgent to assess current situation as experienced by those closely involved and directly affected.

## Introduction

In Portugal, the responsibility for health care is with the Ministry of Health, which coordinates and finances public health care provision, developing health policy and overseeing and evaluating its implementation and managing and regulating the National Health System (NHS). It is also responsible for the regulation, auditing and inspection of private health services providers. Several other bodies contribute with technical support and expertise such as the High Commissariat for Health, the General Inspectorate of Health-related Activities, the General Directorate of Health, the Institute for Health Quality and the Central Administration of the Health System.

The country is divided into five health regions, corresponding to five health administrations. Regional health administrations are responsible for the regional implementation of national health policy objectives and coordinating all levels of health care, working in accordance with principles and directives issued in regional plans and by the Ministry of Health. Their main responsibilities are the development of strategic guidelines; coordination of all aspects of health care provision; supervision of management of primary health care and hospitals; establishment of agreements and protocols with private bodies; and liaison with government bodies, *Misericórdias* (charity institutions), other private non-profit-making bodies and municipal councils. Regional Health Administrations have been appointed responsible for the development of the National Network of Long-Term Integrated Care.

Whilst health care is the responsibility of health authorities with few exceptions and almost universalised in Europe, the same cannot be said regarding social care and home care, which have been heavily decentralised to regional or local government authorities or are in hands of private entities and many times subjected to means as well as needs test. In case of Portugal, the formal provision of social care, personal care and domestic aid is mostly made by private providers, including non-profit and for-profit [1, 2]. Local government involvement has been marginal and confined to the participation in a few specific projects. The number of for-profit actors in the market is increasing but the main providers so far have been the Private Non-profit Institutions of Social Solidarity (IPSS – *Instituições Particulares de Solidariedade Social*), most of the times in the context of protocols established with the Social Security. IPSS offer a number of social care services, including residential home, day care and home support. Meals-on-wheels, personal care, laundry and domestic aid are available from most of them but other services, such as assistance with taking medication or even some health care may also be provided.

There is also a traditional reliance on the family as the first line of social care, particularly in rural areas. However, due to demographic, epidemiologic and cultural changes, many people can no longer rely on such informal care and the demand for social as well as medical care is escalating. As family support has been decreasing, the State considers IPSS a strategic part of the care system and has formally recognised their activity almost 30 years ago (Decree Law n° 119/83). The government, at central or local level, and IPSS may establish cooperation agreements regarding a particular area (Normative Dispatch n° 75/92, Norma I, n° 4). Co-financing by the Social Security depends on social responses developed by IPSS [3]. Protocols are celebrated annually between the ministry responsible for the Social Security and three unions representing IPSS. A typical agreement defines a value per client per month.

A growing number of for-profit entities are entering onto the market. These companies need to have a licence from the Social Security, which implies meeting all the associated requirements. For-profit entities may succeed in making protocols with the State in which case the services they provide are partially financed by the State. The client pays the remaining fraction of the cost. Apparently, the Social Security is demanding a certification from applying companies. This situation is very recent and disparate information has been collected throughout the country.

This paper researches the setting and key changes being made in the Portuguese health and social care systems and in the way they interact. The objective is to enable analysis and discussion of recent initiatives explicitly or more implicitly aiming at creating conditions for increasing integration of care in Portugal. We first introduce key concepts, dimensions and perspectives on integrated care and clearly state the boundaries of care reforms, in order to set the stage of further work. The Methods section explains the mixed methodology used in field work. In the Results section, the first and second subsections are devoted to the description of recent changes introduced in the Portuguese health and social care systems aiming at better care integration. The third subsection depicts and analyses endeavours sparking from joint efforts from different bodies aiming at increasing integration and continuity of care. We try as much as possible to follow the frameworks found in the literature but reforms and initiatives are not easily framed by these more theoretical schemes as we show in the Discussion and Conclusions sections.

## Theoretical background

Integrated care, both as a concept and in practice, has received increasing attention from policy-makers, health professionals and researchers during the last decade. Promoted by the World Health Organisation as a synonymous of coordination of care across diverse professionals, services, organisations and health sectors intervening in the diagnosis, treatment, care, rehabilitation and promotion of health, it is believed to improve the services in relation to access, quality, user satisfaction and efficiency [4] and some evidence seems to confirm at least in part such expectations [5, 6]. The latter are overarching objectives of most health sector reforms, given place to different strategies and policies [7], according to the context in place.

Integration is often categorised in types and several typologies exist. Depending on the author, integration might be: (of) care, (of) clinical team, functional, normative, systemic [8]; functional, organisational, professional, service or clinical, normative, systemic integration [9]; or structural (vertical, horizontal), functional (clinical, information, financial, administrative), normative, systemic integration [10]. Another conceptualisation understands care integration as a process that takes place within relationships between individuals at the microscopic level, clinical integration and functional integration as taking place with respect to the unit of analysis in question (e.g., territory, organisation, region), normative integration as expressing the relationships between the levels and systemic integration (macroscopic level) as ensuring consistency between the unit of analysis and the environment [8]. The latter in part corresponds to the concepts of micro, meso and macro levels of care, to which a system overarching approach is added. However, the specification of which level to consider meso and/or macro depends much on the situation.

Perspectives of integrated care in line with the World Health Organisation definition of health expand the spectrum of participating entities in the network, to include, among others, those providing services related to activities of daily living to citizens temporarily or definitely unable to take care of themselves. They act as key nodes in the network of care, providing important services to those in need and their families, and increasing the efficiency and the efficacy of other providers and of the network as a whole. Therefore, a holistic approach to integrated care needs to consider not only integration inside the health sector [11] but also integration of social services and even the participation of other partners, such as pharmacists [12], informal caregivers and the entire society [13].

Interventions that span multiple, interlocking domains, both in terms of levels and types of integration, would result in better patient outcomes and system-level performance [14]. However, the list of failed efforts and attempts to integrate health and social care in Europe and elsewhere is long [15–17], reflecting the difficulty in overcoming organisational fragmentation of involved providers, and their differences in governance, accountability, management, culture, vision, professionals involved, processes, information systems, status and priorities [15, 18].

A reform is usually understood as a positive change but progress must go further than just any improvement, it must entail “sustained, purposeful and fundamental change” [19]. A health sector reform is a consequence of a rational, planned and evidence-based process; it is not a temporary effort but must have an enduring impact in the system being transformed; and must address significant, strategic dimensions of health systems [19, 20]. Health reform, as any policy reform, takes place in an environment of a dynamic change in health, economic, political and social conditions. In practice, however, it frequently results from political negotiations among the various actors and political stakeholders in the policy decisions [21], and integrated care is no exception [22]. A reform needs to be considered in a country-specific dimension. Differences in the institutional and historical contexts imply that reforms undertaken may not necessarily reflect the same factors and problems. For the success of a care reform, its preparation with a compliance of national guidelines needs to be guaranteed and participation of the society needs to be encouraged [23].

## Methods

We used a mixed methodology in the research we report. First, we conducted a deep literature review and systematisation of knowledge in the area of integrated care that led to creating concept maps helping us to set up a framework for successive work. This framework included the guidelines and the tools used during interviews and focus groups. Then, we interviewed informants at several levels in order to build a list of major recent changes either explicitly addressing integrated care in the country or which had had impact on the integration of care. The interviewees included medical doctors, social workers and decision-makers affiliated to institutions of the health care system (16 informants from 9 health centres/family health units, hospitals or health care trusts), the Portuguese Social Security (9 informants from 9 district units), municipalities (mainly social workers and decision-makers from 9 municipalities), IPSS (9 social workers and 1 decision-maker from 10 institutions) and one medical doctor, responsible for one rehabilitation unit of the RNCCI. Selection of the institutions was made according to geographical criterion and covers the Continental Portugal and the Autonomous Regions of Azores and Madeira. In a third step, we conducted two independent focus groups with researchers from the University of Aveiro and practitioners from health and social care institutions in the District of Aveiro where those reforms were discussed and evaluated regarding their characteristics and a potential impact on the integration of care in Portugal. Results from the focus groups were analysed, confronted and reconciled, giving place to a list of requisites and guidelines that oriented our further search for documentation regarding those reforms, such as decree laws, published monitoring and audit reports and action plans, informative notes issued by cabinets of ministries and information on the websites of the Ministry of Health, the Ministry of Labour and Social Solidarity, the Social Security and the National Network of Long-Term Integrated Care, among others. For readers interested in contextualising this research within more wide scenarios of reforms and evolution of the Portuguese health and social care systems, several works are available in the literature [24–30].

## Results

### Reforms in the Portuguese health care system

In the course of the past 60 years, a number of health care reforms took place in Portugal [26]. Since the early 1990s, the health system has not undergone any key change related to financing policies but many measures have been adopted to improve its performance [31]. Some of them are directly connected to the subject addressed in this work, namely reorganisation of the public network of services, reform of primary care and creation of long-term care networks. Major laws and planned reforms are described in Table 1.

In fact, concerns with integration and continuity of care are present already in the Law on Fundamental Principles of Health, dated to 1990 (Law 48/90). This law states that health services communicate with each other and with social and welfare services on behalf of patients’ interests and that this articulation should be intensively promoted. A health centre constitutes here the first access point to the system, playing an important role for the premise of the continuity of care.

Since 2005, significant changes have been introduced into the structure and organisation of the primary health care system. With Decree Law 88/2005 (reinstating Decree Law 157/1999), health centres were reconfigured and became a framework for various functional units, with a special attention given to a Family Health Unit (USF – Unidade de Saúde Familiar) considered the fundamental element, delivering health services to a population defined through the enrolment list. Advances of the reform in field led to a further definition of the primary health care system constituents. Specification of the status of USF was subsequently defined by Decree Law 298/2007. Decree Law 28/2008 established ACES, public services responsible for health of population in a geographically restricted area, leading to the official extinction of health sub-regions. This should happen with an ordinance making a creation of the last ACES in a given sub-region. An ACES has administrative autonomy and comprises a number of functional units, with organisational and technical autonomy incorporated into health centres frameworks. Instruments guaranteeing articulation between particular functional units of ACES are deemed necessary for the efficient use of common resources.

Hospital care has been subjected to two kinds of reforms, directed to the redefinition of the existing National Health System supply of hospital services on one side, and to the public hospital model, including management rules and payment systems, on the other. Within the first issue, two measures relate directly to the theme of this research: concentration of two or more nearby hospitals under the same management team and the use of Public–Private Partnerships. The other path of reforms is more related to the way the Ministry of Health establishes the payment to the National Health System hospitals and led to two types of hospital statutes: hospitals as public enterprises and hospitals managed by civil service rules. There is, however, no evidence that would permit evaluating the impact of these measures on services integration.

The quest for integration within the health care is not new and several attempts to implementing different models were made during the 1990s. Decree-Law n° 11/93 that approved the Statute of the National Health System established the concept of health care integrated units, composed by hospitals and by personalised groups from health centres of given geographic areas. However, several years after its approval it became evident that the proposed model, previewing inner councils composed exclusively of hospitals’ and health centres’ representatives, could only enable a low level of articulation between hospitals, health centres and other geographically close institutions. Thus, legislation was passed creating local health systems, as panels of complementary resources organised according to geographical and population criteria. Local health systems were integrated into frameworks for hospitals, health centres and other health care provider entities. However, this legislation was never taken into action.

As a result of new proposals, the Local Health Unit of Matosinhos, established by Decree-Law n° 207/99, became the first example of an effective integration of local hospitals and related health centres into a single provider entity. Theoretically, the model aims at enhancing the articulation of one or several hospitals with a determined set of health centres considering geographic proximity, balancing of specialties within a region and assuring medical emergency services to the population [31]. Since then, five new local health units have been implemented [32].

In maternal and child health, an initiative of functional coordinating units has already a 20-year-long experience. Created in 1991 and restructured through Order 12917/98, they have played an important role in what articulation between primary and secondary care in this area concerns [33]. Dispatch n° 9872/2010 fits this legislation to the new reform of the primary health care.

### Reforms in the Portuguese social care system

In Portugal, policy change through law enactment in the area of social care seems to have been rather easy in the past due to the centralisation of the system and the lack of effective social partner participation [25]. A different thing in what concerns care services provision is, however, the State ability to lead involved actors to implement envisaged changes. Therefore, more than listing specific reforms, it is worthwhile to present their results, evident in competing models underlying the structure of the system and the policy agenda since 1974, as stressed in previous research [25].

The first model tends to privilege and reinforce income replacement mechanisms. Under this social insurance model, the State tends to reduce its regulation over non-profit organisations, benefiting from their function as isolating mechanism from social demand regarding access and quality. In a wider version of this logic, the for-profit sector is included in the division of work, supporting the idea that the three sectors should be in competition for service provision and citizen-consumer choice. The most distinct feature of the second model is the combination of income replacement, universal benefits, although means-tested or/and income related, and social and family services. This model does not assume a separation between the State and non-profit organisations and proposes mechanisms where Public–Private Partnerships are created to implement policies and share responsibilities. Conflicts have been reported between the private (particularistic) nature of organisations and the public (universalistic) character of their services [25, p.22].

A recurrent issue is thus the State's ability and will to evaluate and control non-profit organisations. One of the main differences between Portugal and other Southern and Southwest European countries is the high degree of organisation and power of the non-profit sector through strong and powerful peak organisations that participate actively in policy-making.

In this regard, important changes have been initiated in recent years. Following a cooperation programme signed in 2003 between the Ministry of Social Security and Labour and the peak organisations, the Portuguese Institute for Social Security developed a quality model derived from the International Organisation for Standardisation 9001 standard and the Model of Excellence of the European Foundation for Quality Management and adapted to the different types of services provided by social care entities. The certification is to be made by independent entities accredited within the Portuguese Quality System. The Portuguese Institute of Accreditation defines the requirements of accreditation. Three levels of accomplishment have been defined and while certification is not mandatory so far, the revalidation of the protocol with the Social Security depends on meeting certain requirements. On the other hand, the State funding of IPSS services will most probably evolve to payment by service provided to the individual client, instead of payment by client, improving accountability at organisational and system levels and flexibility and agility of the entire network.

### Joint initiatives towards the integration and continuity of care

Before the 1990s, the published references to broad integration efforts and initiatives in Portugal are anecdotal. However, some laws and programmes are sometimes cited as interesting and innovative vehicles that promoted decentralisation of care and connections between primary care, hospitals, the third sector and the community (see Table 2).

In 1963, the first law of mental health (Law n° 2118), unequivocally advanced for its time [34], led to the settlement of mental health centres in every district during the next two decades and to some interesting experiences of outreaching teams from psychiatric hospitals working on the community, an initiative of connection to health centres with significant expansion. Another important step was the settlement of new departments of psychiatry and mental health in general hospitals, which improved access and better coordination with health centres and community agencies [34]. Programmes and structures aiming at psychosocial rehabilitation, including employment support, have been developed. A national mental health information system has been implemented.

Unfortunately, several constraints resulted in deficient implementation and put Portugal behind relatively to other European countries [34]. The Proposal of an Action Plan for the Restructuring and Development of the Mental Health Systems in Portugal for the period 2007–2016 addresses this situation and advances measures to deal with fragmentation and disarticulation, including development of local services networks, better articulation with primary care, with training programmes for general practitioners and improvement of information management, better articulation between health care and social care organisations, aiming at social rehabilitation and integration, and a national network of long-term integrated care for mental health. Concomitantly, the Decree-Law n° 8/2010 launched “*multidisciplinary mental health structures of long-term integrated care, adapted to specific age groups characteristics”* that should function in articulation with Local Services of Mental Health and the National Network of Long-Term Integrated Care. These units might take the form of residential units, socio-occupational units or domiciliary support teams.

The Conjoint Dispatch n° 407/98 from the Ministries of Health and Labour and Solidarity, published in 1998, regulates an articulated intervention of social and continued health care support and defines the ADI). The service has been implemented in some health centres and at times coexisted with services provided by the *Rede Nacional de Cuidados Continuados Integrados*. In some health centres, however, services running smoothly were discontinued by the time the *Rede Nacional de Cuidados Continuados Integrados* started to operate, without being replaced by a similar offer within the network of integrated care, given place to a deterioration of the services provided to the citizens.

Another emblematic initiative created by Conjoint Dispatch of the Ministry of Health and the Ministry of Labour and Social Security, from 1 July 1994, is the PAII. Developed at central and local levels, it aims at promoting a better quality of life for the elderly, at home and in the community, discouraging institutionalisation. The programme targets those aged 65 or more, families, neighbours, voluntaries, professionals and community at large. Its main objectives are to promote the autonomy of elder and dependent people, to support families taking care of them, to establish measures for improving mobility and accessibility, to promote and support training for informal and formal caregivers, professionals, relatives, voluntaries and other people from the community, and prevent isolation and exclusion.

The PAII is then managed by representatives from the Social Security Institute and the General Direction of Health and promoted by the Ministry of Health and the Ministry of Labour and Social Security. Partnerships have been established with health services, Social Security district centres, local government (municipalities and parish councils), the Union of the Portuguese *Misericórdias*, IPSS and other non-governmental organisations, the Portuguese Telecom, the Portuguese Red Cross, security services, fire departments, schools, and voluntary organisations.

Efforts towards a better articulation between the Ministry of Health and the Ministry of Labour and Social Solidarity concerning the System of Technical Aids Support and Financing (Decree-Law n° 93/2009) are also to be reported. The system demonstrates a concern in adapting an offer into new needs and demands.

Long-term care did not make part of the public health agenda for long and, besides family, used to be provided by *Misericórdias*. However, the urgency in decreasing the length of stay in expensive acute care units and the need for ensuring smoother care transitions and simplifying tracking the patient across the systems lead to the creation of the National Network of Long-Term Integrated Care by Law n° 101/2006 within the scope of the Ministry of Health and the Ministry of Labour and Social Solidarity. The National Network of Long-Term Integrated Care builds on partnerships among existing institutions in diverse sectors, integral planning and multidisciplinary practice, aiming at satisfying the needs of citizens with variable degrees of dependency.

The network integrates both objectives of social policy and health policy and operates by establishing partnerships between the public sector, the non-profit sector and the private sector in a model of cooperation and mixed financing. Geographically, the network is organised at three levels of co-ordination, central, regional and local, aiming at improved governance and equity of access. Structurally, inpatient care capacity is the result of protocols established between the network and existing institutions, designated according to the kind of services they provide as Convalescence Unit, Medium Term and Rehabilitation Unit, Long Term and Maintenance Unit, Palliative Care Unit and Day Care and Autonomy Promotion Unit. Maximisation of care in the community, both general health care and palliative care, is one of the goals but the implementation of home support teams in health centres only now is taking off.

Coordination with the primary care reform turned out to imply additional efforts for the network, due to more flexibility on the side of the *Rede Nacional de Cuidados Continuados Integrados* and less flexibility on the side of primary care organisations themselves involved in complex organisational and modernisation processes [35].

The management instruments developed to support the implementation and monitoring of the *Rede Nacional de Cuidados Continuados Integrados*, both at national and regional levels, allow for tracing the activities and evolution of the network's services as well as initiating a close monitoring of results and impacts of the delivered care. Referrals of patients to the network and clinical activity in all inpatient units are registered, since 2008, in a dedicated web-based information system accessible from all the units involved.

## Discussion

Evidence shows that care integration is not absent from policy-making and implementation efforts in Portugal. In fact, the country seems to have been making attempts to accompany some good international practices in this regard, adopting legislation sometimes ahead of its time and piloting innovative frameworks. On the other hand, at least in the last decade, the circumstances in the field have been propitious for the development and delivering of integrated care in the country, namely considering all the conditions that have been created with the launch of the National Network of Long-Term Integrated Care. Yet, until very recently, attempts to go beyond pilot initiatives limited in space and time seem to have been hindered by several reasons, sometimes referenced as mostly operational [31]. Different professional cultures, interests and agendas, diverse sometimes opposite goals and situational aspects are among those diagnosed for long time [1].

Recent reports and studies on the reforms focused in this work show, however, that recurrent higher level issues from different nature seem to be consistently present [25, 34, 36]. As a consequence, few measures reflecting a significant number of regulations have been fully implemented. Mental health services that have been the object of a thoroughly sometimes harsh diagnosis and complete action proposal can be used as a discussion example [34]. Mental health services suffer from serious insufficiencies at the level of accessibility, equity and quality of care [34, 36]. Most part of resources is still concentrated in Lisbon, Coimbra and Porto while services created in several points of the country with excellent conditions are working only to some degree or have units that are not yet open due to lack of professionals who have been concentrated in medical units of major urban centres. The decision to locate the centre of local services in general hospitals, without transforming them in centres of responsibility, hinder any attempt to develop networks of community care. The long waiting lists for consultations, the preferred use of emergency services, the proportion of readmissions occurred without any ambulatory contact suggest problems with continuity of care.

The modest level of resources available in Portugal for mental health is certainly one of the factors hampering the development and improvement of services in this area. However, it seems that inequality in the distribution of mental health services is probably a major cause of the situation. It is also a consequence of the model of management and financing of the services [36], the absence of a national or regional plan and of a clear contractual model, the lack of integration between the information systems of participating entities and of coherent evaluation system at all levels [34].

## Conclusions

Important opportunities to overcome difficulties in integration of care in Portugal seem to be emerging in the context of recent reforms, with the launch of the long-term integrated care programme, the development of Family Health Units, the quest for evaluation and increasing implementation of quality systems and a new drive to pay-for-service. However, these latter huge efforts aiming at better care services in relation to access, quality, user satisfaction and efficiency are themselves being made within increasing stringent conditions and cold financial climate. Therefore, it is urgent and worthwhile to discuss current situation as experienced by those closely involved and directly affected by the system's functioning and try to explain it at the light of information available regarding level of implementation and respective determinants.

## Competing interests

The authors declare that they have no competing interests.

## Authors’ contributions

SS has contributed to conception and design, acquisition of data, analysis and interpretation of data, drafting or revising the manuscript critically for important intellectual content and final approval of the version to be published. NS and PR have contributed to acquisition of data, revising the manuscript and final approval of the version to be published.

## Figures and Tables

**Table 1.**
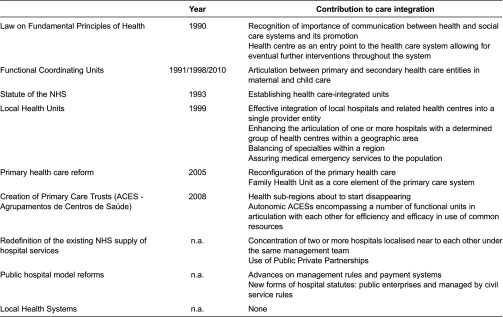
Major laws and planned reforms in the health care system in Portugal

NHS, National Health System.

**Table 2.**
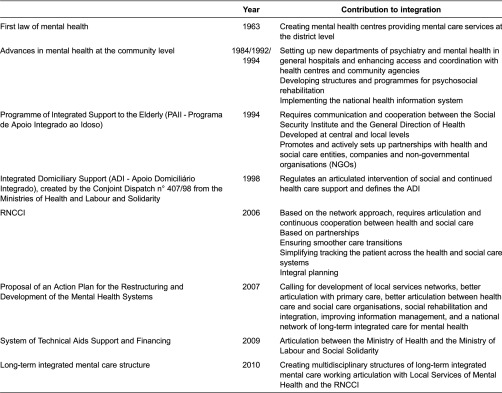
Major initiatives aiming at integrated care in Portugal

ADI, *Apoio Domiciliário Integrado*; RNCCI, *Rede Nacional de Cuidados Continuados Integrados*.
